# Towards global healthy longevity: report from the 1st World Longevity Summit in Kyotango, Japan

**DOI:** 10.1038/s41514-025-00279-0

**Published:** 2025-11-18

**Authors:** Tomoya Kitani, Satoaki Matoba, Yuji Naito, Steve Horvath, Tamotsu Yoshimori, Francis K. L. Chan, Tomoo Matsuda, Stefania Bandini, Hironori Nakagami, Ryuichi Morishita, Yasushi Nakayama, Hitoshi Yaku

**Affiliations:** 1 Department of Cardiovascular Medicine, Graduate School of Medical Science, Kyoto Prefectural University of Medicine, Kyoto, Japan; 2 Department of Human Immunology and Nutrition Science, Graduate School of Medical Science, Kyoto Prefectural University of Medicine, Kyoto, Japan; 3 Altos Labs, Cambridge, UK; 4 Department of Beyond Cell Reborn Research, Graduate School of Medicine, The University of Osaka, Osaka, Japan; 5 Microbiota I-Center (MagIC), Faculty of Medicine, Chinese University of Hong Kong, Hong Kong SAR, China; 6 Mitsubishi Research Institute, Tokyo, Japan; 7 Department of Computer Science, Systems and Communications, University of Milano - Bicocca, Milan, Italy; 8 Department of Health Development and Medicine, Osaka University Graduate School of Medicine, Osaka, Japan; 9 Department of Clinical Gene Therapy, Graduate School of Medicine, Osaka University, Osaka, Japan; 10 Kyotango City Government, Kyotango, Japan; 11 Department of Cardiovascular Surgery, Graduate School of Medical Science, Kyoto Prefectural University of Medicine, Kyoto, Japan

**Keywords:** Health care, Health humanities

## Abstract

Aging societies around the world face increasing challenges in promoting healthy longevity. The 1st World Longevity Summit in Kyotango, Japan, convened experts to discuss advances in aging biology, lifestyle medicine, and community-based health strategies. The joint declaration outlined four pillars for healthy aging: bonds with communication, dietary fiber, physical activity, and “Ikigai”, a sense of purpose. This report highlights representative summit discussions and presents key insights on healthy longevity.

## Global aging and the quest for healthy longevity

The rise of aging societies is a global phenomenon, affecting nations across all continents regardless of economic status^1^. As life expectancy increases and birth rates decline, countries are facing mounting challenges in sustaining healthcare systems, labor forces, and intergenerational cohesion. At the same time, while average life expectancy continues to rise, the gap between lifespan and healthspan, defined as the years lived in good health, remains substantial in many countries^2^. This disparity underscores the urgent need not only to extend life, but also to ensure those additional years are lived with independence, vitality, and dignity. It is in this context that the 1st World Longevity Summit adopted the theme “Extending Healthy Life Expectancy Around the World,” aiming to foster global collaboration toward sustainable and equitable strategies for healthy aging.

Japan which now has one of the highest proportions of elderly individuals in the world. As of 2025, nearly 30% of Japan’s population is aged 65 or older, and this figure is expected to grow further in the coming decades^3^. Within Japan, the Kyotango region in northern Kyoto Prefecture has drawn attention as a potential source of insight into healthy longevity accompanied by strong well-being and happiness, with the centenarian rate nearly three times the national average^4^. Factors such as strong social cohesion, plant-forward dietary habits, daily physical activity, and a rich sense of cultural identity are thought to play a role in supporting healthy longevity. These features make Kyotango a valuable example for exploring strategies to promote healthy aging.

Inspired by these regional characteristics, the 1st World Longevity Summit was held in Kyotango to bring together leading experts from around the world. The summit comprised 28 presentations spanning basic science, clinical medicine, public health, culture, and community initiatives, with the goal of sharing cutting-edge research and practical strategies for healthy aging. While the summit featured many insightful sessions across diverse fields, we summarize the presentations delivered in the three symposia and the two closing lectures. These sessions were selected because they addressed broad, cross-cutting themes in aging biology, lifestyle medicine, and geroscience, and highlighted perspectives on translating scientific advances into community and policy frameworks. Together, these selected presentations highlight the summit’s central theme: that healthy longevity must be pursued through interdisciplinary and globally relevant approaches.

## Epigenetic clocks and the biology of aging

Steve Horvath of Altos Labs delivered a comprehensive presentation on epigenetic clocks, which are mathematical models based on DNA methylation patterns used to estimate age at the molecular level^5^. Originally designed to predict chronological age in human tissues, these clocks have evolved into robust biomarkers for studying biological aging across species. He demonstrated that offspring of centenarians have younger epigenetic ages, underscoring genetic and lifestyle influences on longevity. He also showed that the rate of epigenetic aging varies across organs; in centenarians, the cerebellum and retina age more slowly, while blood and bone age faster. The clocks also detect prenatal development and show full rejuvenation when cells are reprogrammed to induced pluripotent stem cells. Together, these findings highlight the versatility of epigenetic clocks as tools for understanding aging dynamics across the human lifespan.

Horvath next introduced GrimAge, a biological epigenetic clock that incorporates protein biomarkers and smoking history to more accurately predict mortality and disease risk^6^. He discussed how various lifestyle factors influence epigenetic aging. For instance, obesity was shown to accelerate liver aging, whereas exercise and dietary changes resulted in only minimal reductions in biological age. Similarly, liver methylation age did not significantly decrease in the short term following bariatric surgery. Notably, a Swiss clinical trial found that omega-3 supplementation led to a small but statistically significant reduction in biological age, while vitamin D and exercise showed no significant impact^7^. Additionally, analyses showed that Hispanic individuals exhibited biologically younger profiles despite elevated health risks, consistent with the well-documented “Hispanic paradox.” A similar trend was observed in the Tsimane people, an Indigenous population in the Bolivian Amazon, who also displayed unexpectedly low biological ages despite limited access to modern healthcare. Such results demonstrate the clock’s capacity to reflect subtle influences of lifestyle and environment on the aging process.

Horvath also presented the development of a universal mammalian clock, which estimates relative biological age across more than 180 species and enables direct cross-species comparisons^8^. Using this tool, he demonstrated that interventions such as caloric restriction, growth hormone deficiency, and hibernation are associated with slower biological aging, whereas high-fat diets and genetic conditions like Down syndrome accelerate the process. He further emphasized that biological age is tissue-specific and varies depending on the organ in which methylation is measured. For example, conditions such as hypertension were shown to accelerate aging in organs like the liver and kidney. He concluded that epigenetic clocks offer a powerful framework for investigating the biology of aging and hold considerable potential for assessing the effectiveness of health interventions across a wide range of biological and clinical contexts.

## Autophagy and the cellular mechanisms of aging

Tamotsu Yoshimori of Osaka University delivered a comprehensive presentation on autophagy, highlighting it as a fundamental process in maintaining cellular health and regulating aging. He began by explaining the mechanism of macroautophagy, a conserved cellular pathway in which damaged organelles and proteins are enclosed within autophagosomes and then degraded by lysosomes. Although autophagy was first described in the 1950s, its molecular underpinnings remained unclear until the discovery of ATG genes in yeast by Yoshinori Ohsumi^9,10^. This breakthrough transformed the field and was later recognized with a Nobel Prize. Yoshimori, who joined Ohsumi’s laboratory in 1996, contributed to expanding these insights to mammalian systems and helped establish autophagy as a vital mechanism in age-related health and disease.

Yoshimori emphasized autophagy’s essential roles in energy metabolism, protein turnover, and cellular quality control. A major milestone was the identification of LC3 as a key marker of autophagosomes, enabling visualization of the process and establishing LC3 as a standard in the field^11^. He also described the emergence of selective autophagy, including xenophagy (targeting pathogens) and lysophagy (removing damaged lysosomes), both critical to disease resistance and organ integrity. As an example of his recent work, Yoshimori presented findings on Rubicon, a protein his team identified as a negative regulator of autophagy^12^. Rubicon expression increases with age, impairing autophagic function. Reducing Rubicon, especially in neurons, extended lifespan and improved motor function. Rubicon was also found to promote the release of exosomes carrying senescence-inducing microRNAs, suggesting that Rubicon promotes aging not only by impairing autophagy but also by facilitating the intercellular spread of senescence signals. These findings highlight how modulating autophagy may contribute to healthier aging at the cellular level.

To explore potential applications, Yoshimori established a research-oriented company investigating autophagy-based interventions. As one example, his team has studied a traditional fermented tea from Tokushima, which in unpublished animal studies was shown to enhance autophagy and to suggest possible benefits for lifespan and cellular senescence. In addition, the company has developed and piloted a lifestyle program integrating diet, supplementation, aerobic exercise, and sleep optimization, aimed at supporting autophagy. Although clinical validation remains limited, and scalability, generalizability, and long-term safety require careful consideration, these challenges are driving continued innovation and exploration in the field. He closed by stressing the importance of bridging academic insights with real-world impact, and introduced the Japan Autophagy Consortium^13^, which fosters industry collaborations, product certification, and public education to raise awareness about autophagy’s significance in healthy aging.

## The gut microbiome and the science of healthy aging

Francis Chan of the Chinese University of Hong Kong emphasized the gut microbiome as a central regulator of healthy aging. As Co-Director of the Microbiota I-Center (MagIC), he integrates artificial intelligence, bioinformatics, and clinical data to explore microbiota-driven interventions for aging and disease^14^.

Chan described how the gut microbiome develops from birth, initially shaped by maternal transmission and later by diet and environment. As people age, intestinal function declines, microbial composition changes, and inflammation increases^15^. These shifts contribute to immunosenescence, cognitive decline, metabolic disorders, and increased vulnerability to infections. He stressed the importance of maintaining gut barrier integrity and microbial balance to support systemic health. Interestingly, centenarians do not retain a youthful microbiome. Instead, they exhibit a reduction in core microbial species and a greater diversity of minor beneficial taxa, suggesting a unique microbial adaptation associated with advanced age. In healthy gut aging, a decline in mucus-degrading bacteria such as Bacteroides is counterbalanced by beneficial species like Akkermansia and Bifidobacteria, which help preserve mucosal integrity. In contrast, unhealthy gut aging is marked by persistent Bacteroides dominance, which may worsen barrier damage.

Expanding on how gut microbes influence aging, Chan discussed three key microbial metabolites: butyrate, exopolysaccharides, and spermidine. These compounds may promote longevity by modulating epigenetics, mitochondrial function, and autophagy. He also introduced four categories of microbiome-based aging biomarkers: microbial diversity, taxonomic composition, metabolite profile, and functional output^16^. At the same time, he cautioned that current microbiome-based interventions, such as probiotics and fecal microbiota transplantation (FMT), have shown inconsistent efficacy in older adults. In some cases, FMT from young donors may even cause harm by introducing mucus-degrading microbes into vulnerable recipients.

Next, Chan emphasized that infancy offers a critical window for shaping lifelong health. He introduced a longitudinal birth cohort study across Hong Kong and mainland China, tracking families from pregnancy through early childhood. Findings from the cohort revealed that infants born during the COVID-19 pandemic exhibited delayed microbial maturation and a higher risk of allergies, likely due to increased disinfectant use^17^. The study also found that maternal gestational diabetes was associated with altered infant microbiota and increased head circumference in male infants^18^. His team also identified gut microbiota patterns in infancy that were highly predictive of later autism development^19^. This work was subsequently acknowledged by the U.S. FDA as a notable advance in diagnostics. Building on diagnostic and epidemiological findings, Chan’s team evaluated a synbiotic formulation combining probiotics and prebiotics, designed to influence microbial metabolites relevant to brain function. In a double-blind, randomized trial with more than 500 long COVID patients, the synbiotic intervention demonstrated significant improvements in symptoms such as fatigue, memory, and concentration^20^. These findings suggest that modulation of the human gut microbiome can lead to neurocognitive improvement possibly via the gut–brain axis. Although further studies are needed to confirm generalizability, this body of work illustrates how microbiome-targeted strategies may represent a promising avenue for therapeutic intervention.

Taken together, his research highlights the microbiome as a promising and underutilized pathway to promote lifelong health. He closed with a personal message, emphasizing that although aging is inevitable, maintaining a healthy gut, curiosity, and a sense of purpose can support both physical and mental vitality.

## Designing longevity societies through community engagement and digital innovation

In a closing session, Tomoo Matsuda, Research Director of the Mitsubishi Research Institute, and Stefania Bandini of the University of Milano-Bicocca offered perspectives that broadened the summit’s focus from biological mechanisms to societal frameworks for longevity.

Matsuda presented the concept of a “Platinum Society,” which reimagines older adults as active contributors to society^21^. Contrasting the negative connotations of a “Silver Society,” he proposed a framework in which the elderly bring value to citizens, businesses, local governments, and academia. Drawing on real-world data from regional pilot programs, he showed that community-based initiatives fostering social participation, daily activity, and intergenerational ties could contribute to a decline in long-term care needs, even amid an aging population.

Among various initiatives, he introduced “Exadon” as a representative example of community-based programs aligned with the “Platinum Society” vision^22^. This program, which combines exercise, traditional Japanese taiko drumming, and group interaction, is designed to fosters both physical well-being and social connection among older adults. He also highlighted community practices such as multi-use community centers where seniors mentor children, illustrating how intergenerational engagement can benefit society. Because such community practices cannot easily be evaluated with the rigor of controlled trials, their assessment inevitably has limitations; nevertheless, they play a valuable role in promoting activity, connectedness, and participation in local settings. Finally, Matsuda advocated for policy innovations such as care credits and “second compulsory education” for seniors to support lifelong participation in society. He emphasized that such approaches can help reframe longevity as a positive and inclusive force across all sectors of society.

Following this, Bandini presented how digital tools and geographic data can support aging populations, especially in rural and mountainous regions. She began by introducing AGE‑IT, a major national research initiative in Italy that addresses the social and technological challenges of an aging society^23^. She illustrated the challenges facing Italy’s “inner areas,” which comprise 60% of national land and house 22% of the population. These regions, much like rural Japan, struggle with depopulation, limited infrastructure, and dependence on informal care networks. While traditional diets, strong social ties, and clean environments in these areas support healthy aging, the outmigration of younger generations has led to social fragmentation, loss of traditional knowledge, and declining access to essential services.

To design infrastructure that meets the needs of aging populations in rural areas, Bandini argued that walkability is an essential factor, especially in mountainous terrain^24^. Drawing on case studies, she explained how Geographic Information Systems (GIS), AI, and IoT technologies can help identify local vulnerabilities, such as limited access to medical care, nutritious food, and caregiving services, and support the design of targeted interventions^25^. These findings underscore the importance of integrating digital innovation with local needs and conditions to foster age-friendly environments in rural communities.

Together, the two speakers underscored the importance of viewing population aging not as a burden, but as a chance to redesign society with a balance of innovation and compassion, and to align technological solutions with human dignity.

## Declaration of the 1st World Longevity Summit—toward a global society of healthy and fulfilling longevity

**Fig. 1:**
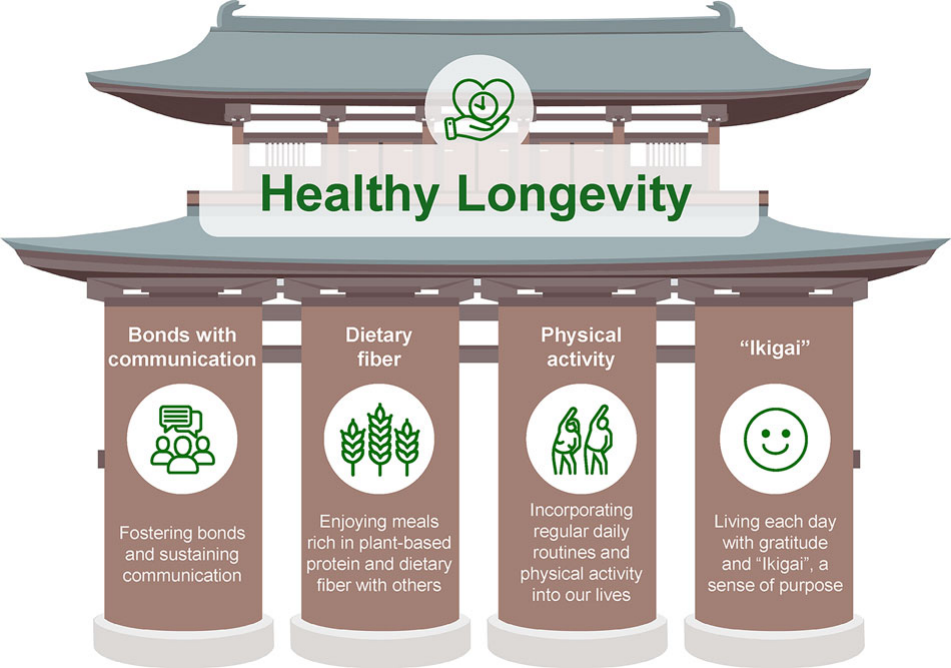
Four pillars of the Declaration from the 1st World Longevity Summit. The illustration conceptualizes how fostering bonds and communication, plant-based communal dining rich in dietary fiber, daily routines with physical activity, and living with gratitude and “Ikigai” collectively support the vision of healthy longevity.

At the conclusion of the 1st World Longevity Summit, both President Hitoshi Yaku of Kyoto Prefectural University of Medicine and Mayor Yasushi Nakayama of Kyotango City delivered closing remarks, which were followed by the joint declaration. Drawing on the discussions at the summit and prior studies, a growing body of evidence indicates that social connectedness is associated with lower mortality risk^26–28^; plant-based diets are linked to better health outcomes and reduced chronic disease risk^29–31^; regular physical activity helps preserve function and delay decline^32,33^; and, finally, having a sense of purpose in life has been prospectively associated with greater longevity^34,35^. These insights were synthesized into the four pillars of the declaration (Fig. 1), setting forth a shared vision and guiding principles for building a future in which all individuals can enjoy physical health, emotional happiness, and overall well-being with dignity:

1. Fostering bonds and sustaining communication
  Human connection is the foundation of mental and physical well-being. We commit to creating a society where people support and engage with each other across generations and communities.
2. Enjoying meals rich in plant-based protein and dietary fiber with others
  Sustainable and healthy eating—centered on plant-based protein and dietary fiber—nurtures both our bodies and the planet. We value sharing meals with others and preserving local food cultures.
3. Incorporating regular daily routines and physical activity into our lives
  A rhythmic lifestyle and moderate daily physical activity help maintain youthfulness of mind and body, forming the basis of healthy longevity.
4. Living each day with gratitude and “Ikigai”, a sense of purpose
  Spending our time with meaning enriches life’s quality and brings forth lasting happiness.

The messages delivered by President Yaku and Mayor Nakayama, together with the four guiding principles of the declaration, underscored the importance of combining scientific insight with cultural values and community-based action to advance a society of healthy and fulfilling longevity.

## Conference summary and conclusion

To provide readers with a structured overview of the conference content, we synthesized all presentations into four thematic areas: (1) deciphering molecular and cellular mechanisms of aging, (2) linking microbiome, metabolism, and nutrition to healthy longevity, (3) deriving insights from population-based and regional studies, and (4) fostering community, lifestyle, and cultural practices for healthy aging. For each thematic area, the principal insights, translational opportunities, and current limitations are summarized in Table 1.

**Table 1:** Key thematic areas of the 1st World Longevity Summit: principal insights, translational opportunities, and current limitations

Thematic area	Principal insights	Translational opportunities	Current limitations
Deciphering molecular and cellular mechanisms of aging	Research emphasized conserved biological hallmarks of aging, identifying epigenetic regulation, autophagy, cellular senescence, and redox balance as central to longevity.	Development of biomarkers for aging and therapeutic strategies targeting fundamental mechanisms of aging.	Evidence remains largely preclinical or preliminary, with limited clinical validation and uncertainties about safety and scalability.
Linking microbiome, metabolism, and nutrition to healthy longevity	Presentations highlighted the interplay of gut microbiota, metabolic regulation, and dietary factors as interconnected drivers of resilience and healthspan.	Microbiome-based interventions, metabolic modulation, and dietary strategies to promote healthy aging.	Many findings are preliminary or context-specific; replication, long-term efficacy, and generalizability remain open challenges.
Deriving insights from population-based and regional studies	Cohort and regional data provided empirical evidence on how demographic, lifestyle, and environmental factors shape patterns of aging and longevity.	Application of cohort findings to public health surveillance, risk stratification, and evidence-based policy design.	Findings may not be generalizable beyond the studied populations, and observational designs limit causal interpretation.
Fostering community, lifestyle, and cultural practices for healthy aging	Community initiatives and cultural practices demonstrated how social engagement, purposeful living, shared meals, and daily routines can be mobilized to promote healthy aging.	Development of scalable community programs and cultural frameworks that integrate lifestyle, social connection, and sense of purpose into healthcare and community systems.	Evidence is often observational and context-dependent, and rigorous evaluation of sustainability and impact remains limited.

The principles outlined in the declaration reflect the core message of the summit and present a forward-looking vision in which aging is approached with optimism, dignity, and a focus on well-being. Over 4 days of dialog in Kyotango, researchers, innovators, policymakers, and community members explored diverse approaches to achieving healthy and fulfilling longevity. Through the exchange of scientific findings and real-world experience, the summit highlighted how the challenges of aging can be reframed as opportunities for progress. As the declaration affirmed, the summit is not a conclusion, but a call to action for building a society of healthy longevity. We further believe that the pursuit of healthy longevity must be inclusive, ensuring that approaches are relevant and accessible across diverse regions and populations worldwide. Sustained collaboration across science, culture, communities, and government will be essential to realizing a world where everyone, regardless of region or background, can live longer lives with health, happiness, well-being, dignity, and purpose.

## Data Availability

No datasets were generated or analyzed in the preparation of this meeting report.
